# Exploring frailty in the context of HIV and aging in Kazakhstan: findings from a pilot cross-sectional study

**DOI:** 10.1186/s12889-026-26548-5

**Published:** 2026-02-07

**Authors:** Balnur Iskakova, Deborah Gustafson, Aigerim Alimbekova, Nursultan Nurzhigitov, Anarkhan Nurkerimova, Gulmira Kalzhanbayeva, Gulnara Nugumanova, Ademi Sarsembiyeva, Jack DeHovitz, Zhamilya Nugmanova

**Affiliations:** 1 Department of Health Policy and Management, Asfendiyarov Kazakh National Medical University, School of Public Health, Tole Bi Street 94, Almaty, 050000 Kazakhstan; 2 Department of Neurology, SUNY Downstate Health Sciences University, New York City, NY USA; 3 Almaty Center for AIDS Prevention and Control (ACAPC), Almaty, Kazakhstan; 4 Department of Epidemiology, Asfendiyarov Kazakh National Medical University, School of Public Health, Almaty, Kazakhstan; 5 Department of Medicine, SUNY Downstate Health Sciences University, New York City, NY USA

**Keywords:** Frailty, People living with HIV, Preliminary findings, Central asia, Kazakhstan, Aging with HIV

## Abstract

**Background:**

Frailty is increasingly recognized as an important morbidity among people living with HIV (PLHIV). However, no published data exist on its prevalence or determinants in Kazakhstan or Central Asia. This pilot study aimed to provide preliminary estimates of frailty prevalence and its associated factors among PLHIV in Almaty, Kazakhstan.

**Methods:**

We conducted a cross-sectional pilot study at the Almaty Centre for AIDS Prevention and Control. A total of 100 PLHIV aged 40 years and older were recruited during routine clinical visits from a previous pilot study on cardiovascular health. Frailty was assessed via the Fried frailty Phenotype that included slow gait speed, weak grip strength, exhaustion, low physical activity, and unintentional weight loss. Participants meeting three or more criteria were classified as frail, and those meeting two or more criteria were classified as prefrail/frail. Logistic regression models were used to examine associations of prefrailty/frailty with HIV-related factors, multimorbidity (≥ 3 chronic conditions other than HIV), depressive symptoms, HIV-related stigmas, and expectations regarding aging.

**Results:**

The participants had a mean age of 50 years (SD 7.9), and 58% were male. The prevalence of frailty was 13%, whereas 40% met the criteria for prefrailty/frailty. Multimorbidity (adjusted odds ratio [AOR] 3.44, 95% confidence interval [CI] 1.40–8.79), depressive symptoms (AOR 1.09, 95% CI 1.02–1.19), internalized HIV-related stigma (AOR 1.25, 95% CI 1.05–1.56), and clinic-based HIV stigma (AOR 1.57, 95% CI 1.18–2.10) were associated with greater odds of prefrailty/frailty. Neurological, endocrine/metabolic, and noninfectious respiratory conditions were also significantly associated with the outcome. HIV-specific indicators such as the CD4 count and viral load were not significantly associated.

**Conclusions:**

Frailty is present among middle-aged and older PLHIV in Kazakhstan. Psychosocial factors and multimorbidity were more strongly associated with frailty than were HIV-specific indicators. As a pilot study with a modest sample size, these findings should be interpreted as preliminary but emphasize the need for larger-scale studies and interventions addressing frailty among PLHIV in the region.

## Background

The global population is undergoing a demographic transition characterized by increasing life expectancy and declining fertility rates. As a result, societies worldwide are experiencing older population demographics. According to national forecasts, the number of individuals aged 65 years and older in Kazakhstan is projected to increase from 1.4 million in 2019 to approximately 3.4 million by 2030, increasing their share of the total population from 7.5% to over 14% [1]. This demographic trend signals an urgent need to strengthen health and social support systems to address the growing prevalence of age-related conditions [2].

In parallel, the aging of another vulnerable population, people living with HIV (PLHIV), presents a complex and under-recognized public health issue. Advances in antiretroviral therapy (ART), combined with earlier diagnosis, have extended the life expectancy of PLHIV [3, 4]. Furthermore, PLHIV continue to face a disproportionately greater burden of age-related comorbidities and geriatric syndromes at comparatively younger ages than other individuals do [5–7]. Studies have shown that these individuals may experience clinical manifestations of aging 5–10 years earlier than people without HIV [5, 6]. This phenomenon is also known as “accelerated aging”, which may be due to chronic immune activation, persistent systemic inflammation, long-term ART toxicity, and HIV-related immune dysregulation [8]. Additionally, non-HIV-related risk factors such as financial instability and poor access to adequate healthcare, stigma, and substance use may further exacerbate the vulnerability of PLHIV to older age-associated conditions [6].

One of the most significant and multifaceted conditions associated with older age is frailty. Frailty is a geriatric syndrome characterized by decreased physiological reserve and increased vulnerability to stressors, resulting in a greater risk of adverse health outcomes, including noncommunicable diseases, hospitalization, functional decline, and mortality [9]. While traditionally observed among people aged 65 years and older, studies have expanded the scope of frailty to include younger populations, particularly those with chronic health conditions such as HIV. Studying prefrailty is also important for preventing or delaying the onset of overt frailty [10]. Both frailty and prefrailty can be measured via the Fried Frailty Phenotype (FFP) definition, which comprises five criteria: self-reported unintentional weight loss, exhaustion, low physical activity, and objective measures of weakness and slowness [11]. Individuals are determined to be frail if they meet three or more of these criteria, whereas those meeting at least two of them are considered prefrail. Among PLHIV, frailty prevalence is reported to range from 5% to 20%, depending on age, treatment status, and comorbid conditions [12]. This is notably higher than the 5–10% prevalence of frailty reported in the general population aged *≥* 65 years [12–16].

Studying frailty is important because of its associations with adverse health outcomes, such as multimorbidity, hospitalization, disability, and mortality, particularly among middle-aged and older PLHIV. Despite growing international attention, frailty remains unexplored in Kazakhstan, both in the general population and among PLHIV. Most existing studies of aging with HIV in Kazakhstan have focused on broader topics such as quality of life, healthcare access, or specific disease burdens, with little emphasis on geriatric syndromes or age-related vulnerabilities [17–19]. The limited body of research may be due to the country’s relatively recent demographic transition to an older society, as well as a lack of specialized geriatric training and infrastructure.

In this study, we attempted to generate preliminary data on the prevalence of frailty among PLHIV in Almaty, Kazakhstan, via both objective and subjective measures. We also explored the demographic, clinical, physical, and behavioral factors contributing to frailty in this population, aiming to provide initial evidence in Kazakhstani and Central Asian context.

## Methods

### Study design and sample

This study builds upon a previous pilot investigation on cardiovascular health in PLHIV. In 2023, 150 participants from the Almaty Centre for AIDS Prevention and Control (ACAPC) consented to be re-contacted for follow-up research. ACAPC provides HIV services to both urban and rural residents of Almaty, which has the highest HIV prevalence after Eastern Kazakhstan, Karaganda, and Pavlodar [17]. Data collection was conducted during routine visits to the ACAPC in 2024 and included subjective and objective measurements. Participants aged *≥* 40 years and older were recruited from the 2023 pilot cohort (*n* = 100).

### Data collection and measures

Data were collected via physician-administered questionnaires using validated and standardized methods at the ACAPC. Before data collection, the ACAPC staff received training on the administration of both objective and subjective measures of the FFP and other study variables. All the surveys were conducted in Russian language. Study data were subsequently stored and managed using the Centers for Disease Control and Prevention KoBo Toolbox platform [20].

This pilot study included subjective and objective data collection such as Short Physical Performance Battery (handgrip strength and gait speed) and blood sample collection to assess HIV-related factors. Anthropometric data, such as body height and weight, were obtained from the previous pilot investigation.

### Outcome variable: fried frailty phenotype (FFP)

Frailty was assessed via the FFP, which includes five components: (1) unintentional weight loss, (2) exhaustion, (3) low physical activity, (4) impaired mobility (slowness), and (5) reduced grip strength (weakness) (Table 1). Mobility was assessed via 4-meter gait speed, with frailty thresholds defined by the U.S.-based Cardiovascular Health Study (CHS) criteria [11]. Grip strength was measured using a hand-held dynamometer, with thresholds determined based on CHS cut-offs and adjusted for body mass index (BMI), which was calculated as body weight in kilograms divided by height in meters squared (kg/m²). Low physical activity was defined as self-reported inability to engage in work, household tasks, school, or childcare over the past four weeks. Exhaustion was assessed through self-reported difficulty in performing daily activities due to physical health limitations. Unintentional weight loss was defined as a self-reported loss of ≥ 5 kg since the previous pilot study visit. Participants meeting three or more FFP criteria were classified as frail (FFP = 3–5), whereas those meeting at least two criteria were categorized as prefrail/frail (FFP = 2–5). The prefrail/frail category was used as an outcome variable in multivariable regression models.

**Table 1:** The fried frailty phenotype and operationalization of each frailty component

Frailty Component	FFP criteria
Unintentional weight loss	Since your last visit, have you had unintentional weight loss of at least 10 pounds?
	a. Yes
	b. No
Exhaustion	During the past 4 weeks, as a result of your physical health, have you had difficulty performing your work or other activities?
	a. All of the time
	b. Some of the time
	c. None of the time
Low physical activity	Does your health now limit you in vigorous activities, such as running, lifting heavy objects, or participating in strenuous sports?
	a. Limited a lot
	b. Limited a little
	c. Not limited at all
Slowness (same as CHS)	4-meter walk at usual pace:
	a. ≥ 6.13 s for height ≤ 1.59 m
	b. ≥ 5.25 s for height > 1.59 m
	c. All other values
Weakness (same as CHS)	Grip strength measured by Jamar dynamometer:
	a. ≤ 17.0 kg for BMI ≤ 23 kg/m
	b. ≤ 17.3 kg for 23 < BMI ≤ 26 kg/m
	c. ≤ 18.0 kg for 26 < BMI ≤ 29 kg/m
	d. ≤ 21.0 kg for BMI > 29 kg/m
	e. All other values

### Covariates

#### HIV-related variables

The HIV-related factors included the CD4 cell count, HIV viral load, and duration of HIV infection. The CD4 + T-cell count was categorized into three groups: >500, 200–500, and < 200 cells/mm³. The HIV viral load was classified as ≤ 1000 or > 1000 copies/mL on the basis of thresholds established in the literature [21]. HIV duration was treated as a continuous variable, representing the number of years since clinical diagnosis.

#### Multimorbidity and depressive symptoms

The presence of multimorbidity was based on self-reported diagnoses of chronic conditions other than HIV. Participants were classified as having multimorbidity if they reported three or more of the following conditions: stroke, heart disease, lung disease, diabetes, hypertension, hypercholesterolemia, traumatic brain injury, thyroid disease, liver disease, or bone disease. This variable was dichotomized for the analysis (yes vs. no). Depressive symptoms were assessed using the Patient Health Questionnaire-9 (PHQ-9), which measures the frequency of depressive symptoms over the past two weeks based on DSM-5 criteria [22]. The PHQ-9 consists of nine items, each scored from 0 (not at all) to 3 (nearly every day), yielding a total score ranging from 0 to 27. Higher scores indicated more depressive symptoms.

#### HIV-related stigma measures

Two types of HIV-related stigma were assessed: internalized stigma and stigma experienced within clinics that provide HIV care. HIV-related internalized stigma was measured using a six-item scale based on the Stigma Index 2.0, which has been previously validated and used in multiple countries, including Kazakhstan (e.g., “My HIV infection makes me feel dirty.”) [23, 24]. The responses consisted of binary options such as “disagree” and “agree” and were summed to generate a total score ranging from 0 to 6 [24]. Higher scores indicated greater internalized stigma. Similarly, clinic-based HIV stigma was assessed using a seven-item scale, with higher scores reflecting greater perceived stigma in healthcare (e.g., “You were denied medical services due to your HIV status.”).

#### Expectations regarding aging

Expectations regarding aging were assessed using the Expectations Regarding Aging (ERA-12) scale, which consists of 12 items addressing perceptions of physical health, mental health, and cognitive function later in life. An example item is “When people get older, they need to lower their expectations of how healthy they can be.” Response options ranged from “Definitely false” to “Definitely true”. The final scores were transformed to a 0–100 range, with higher scores indicating more negative expectations regarding aging [25].

Educational attainment was classified into three categories: “High or postgraduate,” comprising individuals with completed undergraduate or graduate degrees; “Specialized secondary,” including those with vocational secondary education or incomplete higher education; and “Up to secondary,” referring to participants who had not completed secondary schooling. Employment status was classified as “Employed” for those working full-time (≥ 40 h per week), part-time (< 40 h per week), or any form of paid employment; “Unemployed” for those actively seeking work or without employment; and “Other” for individuals who were retired, disabled, or reported alternative employment situations. Living arrangements were dichotomized into “Stable housing,” which included individuals residing in their own homes or with relatives, and “Unstable/vulnerable housing,” which included those living in shelters, on the street, or in assisted housing.

Non-communicable diseases (NCDs) were classified into six major categories based on self-reported chronic conditions other than HIV. Cardiovascular conditions included heart disease, hypertension, and hypercholesterolemia. The neurological conditions included stroke, traumatic brain injury, and dementia. Endocrine and metabolic conditions included diabetes, thyroid disease, vitamin deficiencies, and a body mass index (BMI) ≥ 30. Renal and hepatic conditions included kidney and liver diseases. Respiratory conditions, including noninfectious lung diseases and musculoskeletal conditions, were defined by a history of bone fractures in this sample.

### Statistical analysis

All the statistical analyses were performed on R statistical package (version 4.4.1) (R Core Team, 2024) [26]. Descriptive statistics are presented as frequencies and percentages for categorical variables and as the means with standard deviations for continuous variables. Differences between male and female participants were assessed via Chi-square tests. Due to the low prevalence of frailty (FFP = 3–5), we used the dichotomized prefrail/frail (FFP = 2–5) variable as an outcome in the regression models. We initially examined the associations between frailty and each covariate via bivariate logistic regression. Potential covariates with *p* values < 0.10 were considered for inclusion in the multivariable logistic regression models. Three multivariable logistic regression models were constructed: (1) HIV-related variables, (2) HIV stigma measures and expectations regarding aging, and (3) multimorbidity and depressive symptoms. All the models were adjusted for age and sex. Multicollinearity was assessed using the Variance Inflation Factor (VIF), with values below five considered acceptable. Each model provided corresponding adjusted odds ratios (AORs), confidence intervals (CIs), and *p* values. The final multivariable logistic regression model included covariates that remained significant across three multivariable logistic regression models (*p* value < 0.05).

## Results

### Sample characteristics

The study sample consisted of a greater proportion of males (58%), with a mean age of 50 years (SD = 7.87). As shown in Table 2, approximately half of the participants (41%) demonstrated preserved immune function (CD4 > 500 cells/mm³), whereas 16% were severely immunosuppressed (CD4 < 200 cells/mm³). A majority of the samples (88%) exhibited low viral replication, defined as ≤ 1000 copies/mL. With respect to comorbidities, 26% of the sample reported having infections other than HIV, with hepatitis C virus (HCV) being the most prevalent (Table 3). Additionally, 38% of the participants had multimorbidity at the time of the study, and the majority of them had cardiovascular and renal-hepatic conditions (33%). The mean internalized HIV stigma score was higher (M = 2.95, SD = 2.34) than the clinic-based HIV stigma (M = 1.24, SD = 1.79). Overall, most descriptive variables did not differ by sex. However, the prevalence of HCV infection was significantly higher among males compared with females (34% vs. 14%, *p* = 0.003). In addition, the presence of multimorbidity was suggested to be more common among males than females (*p* = 0.06).

**Table 2:** Descriptive statistics of sociodemographic characteristics by sex (*N* = 100; *N* = 42 females; *N* = 58 males)

Variables	All *N* (%)		Females; *N* (%)	Males *N* (%)	*p* value
Frailty Phenotype (FFP = 3–5)		13 (13)	6 (14)	7 (12)	0.98
Pre-Frailty or Frailty Phenotype (FFP = 2–5)		40 (40)	16 (38)	24 (41)	0.90
Covariates					
Age category (years)					
40–49		56 (56)	28 (67)	28 (48)	0.17
50–59		31 (31)	11 (26)	20 (34)	
60–69		9 (9)	3 (7)	6 (10)	
≥ 70		4 (4)	0 (0)	4 (7)	
Education					
High and postgraduate		25 (25)	10 (24)	15 (26)	0.39
Specialized secondary		16 (16)	9 (21)	7 (12)	
Up to secondary		59 (59)	23 (55)	36 (62)	
Employment					
Employed		39 (39)	18 (43)	21 (36)	0.28
Other		22 (22)	6 (14)	16 (28)	
Unemployed		39 (39)	18 (43)	21 (36)	
Living arrangement					
Stable housing		88 (88)	36 (86)	52 (90)	0.77
Unstable/vulnerable housing		12 (12)	6 (14)	6 (10)	
Smoking					
Not at all		14 (14)	8 (19)	6 (10)	0.18
Some days		21 (21)	11 (26)	10 (17)	
Everyday		65 (65)	23 (55)	42 (72)	
CD4 cell count (cells/mL)					
> 500		41 (41)	17 (40)	24 (41)	0.98
500 − 200		43 (43)	18 (43)	25 (43)	
< 200		16 (16)	7 (17)	9 (16)	
HIV Viral Load (copies/mL)					
≤ 1000		88 (88)	39 (93)	49 (84)	0.36
> 1000		11 (11)	3 (7)	8 (14)	
Having more than one infection		26 (26)	11 (26)	15 (26)	1.00
Infectious diseases					
HCV		26 (26)	6 (14)	20 (34)	0.003
HBV		4 (4)	1 (2)	3 (5)	0.61
TB		13 (13)	8 (19)	5 (9)	0.55
Syphilis		3 (3)	1 (2)	2 (3)	1.00

**Table 3:** Descriptive statistics of Multimorbidity and depressive symptoms by sex (*N* = 100; *N* = 42 females; *N* = 58 males)

Variables	All *N* (%)	Females *N* (%)	Males *N* (%)	*p* value
Multimorbidity (having 3 or more chronic conditions other than HIV)	38 (38)	11 (26)	27 (47)	0.06
NCDs by Classification				
Cardiovascular conditions	33 (33)	10 (24)	23 (40)	0.15
Neurological conditions	29 (29)	8 (19)	21 (36)	0.10
Endocrine and metabolic conditions	18 (14)	8 (19)	10 (17)	1.00
Renal and hepatic disorders	33 (33)	11 (26)	22 (38)	0.30
Respiratory conditions	17 (17)	4 (10)	13 (22)	0.11
Musculoskeletal conditions	14 (14)	9 (21)	5 (9)	0.12
PHQ-9 depressive symptom score categories				
None to minimal 0–4	46 (46)	17 (41)	29 (50)	0.80
Mild 5–9	33 (33)	15 (36)	18 (31)	
Moderate 10–14	12 (12)	6 (14)	6 (10)	
Severe 15–19	9 (9)	4 (10)	5 (9)	

### Prevalence and associations with frailty phenotype

The prevalence rates of frailty (FFP = 3–5) and the prefrailty/frailty phenotype (FFP = 2–5) in this study were 13% and 40%, respectively. Table 4 presents the results from the multivariable logistic regression models that were used to explore factors associated with the prefrail/frailty phenotype (FFP = 2–5). HIV-related clinical markers were not significantly associated with the prefrailty/frailty phenotype; however, the observed associations suggested higher odds of prefrailty/frailty among participants with higher HIV viral load and lower CD4 counts, which may reflect limited statistical power.

**Table 4:** Multivariable logistic regression models of the prefrailty/frailty phenotype (FFP = 2–5) and covariates adjusted for sex and age (*n* = 100)

	Model 1 AOR (95% CI)	Model 2 AOR (95% CI)	Model 3 AOR (95% CI)	Final model AOR (95% CI)
CD4 cell count (cells/mm³)				
> 500	Ref.			
500 − 200	1.37 (0.53, 3.51)			
< 200	2.66 (0.80, 9.13)			
HIV Viral Load (copies/mL)				
≤ 1000	Ref			
> 1000	1.40 (0.38, 5.04)			
Other infections				
No	Ref.			
Yes	0.73 (0.26, 1.94)			
Stigmas				
HIV-related internalized stigma		1.25 (1.05,1.56)*		1.30 (1.07, 1.63)*
HIV stigma in healthcare		1.57 (1.81, 2.13)**		1.42 (1.08, 1.92)*
ERA-12 total score		0.99 (0.97,1.02)		
Multimorbidity				
No			Ref.	Ref.
Yes			3.44 (1.40, 8.79)**	2.93 (1.07, 8.40)*
Depressive symptoms				
(overall scores 0–27)			1.09 (1.03, 1.19)*	1.09 (1.01, 1.20)*

* *p* < 0.05

**p < 0.01

In contrast, several other factors were significantly associated with frailty. Specifically, multimorbidity (AOR = 3.44, 95% CI = 1.40,8.79; *p* = 0.002), depressive symptoms (AOR = 1.09, 95% CI = 1.02, 1.19; *p* = 0.02), HIV-related internalized stigma (AOR = 1.25, 95% CI = 1.05, 1.56, *p* = 0.01) and clinic-based HIV stigma (AOR = 1.57, 95% CI = 1.81, 2.1, *p* = 0.01) were positively associated with the prefrail/frailty phenotype (FFP = 2–5). These associations remained significant in the final model. In other words, study participants with prefrailty/frailty phenotype (FFP = 2–5) had higher odds of experiencing internalized and clinic-based HIV stigma, to report multimorbidity, and to have higher depressive symptom scores than others in this sample. The multicollinearity test results indicated that all the VIF values were less than 1.50, suggesting that multicollinearity was not a concern in the final models.

We further investigated the associations between specific NCDs and the frailty/prefrailty phenotype (FFP = 2–5) by conducting logistic regression models on five NCD classifications (presented in Table 3), adjusting for sex and age. Individuals with neurological conditions (AOR = 3.41, 95% CI = 1.24, 9.87, *p* = 0.02), endocrine and metabolic conditions (AOR = 3.99, 95% CI = 1.39, 12.66, *p* = 0.01), and respiratory conditions (AOR = 3.59, 95% CI = 1.20, 11.71, *p* = 0.02) had higher odds of being prefrail/frail (FFP = 2–5).

## Discussion

This study aimed to provide preliminary evidence on frailty prevalence and associated factors among PLHIV in Almaty, Kazakhstan. We found that approximately 13% of the participants met the criteria for frailty (FFP = 3–5), and a significantly greater proportion of the study participants were classified as prefrail/frail (FFP = 2–5). The key factors associated with prefrailty/frailty in our cohort included multimorbidity, depressive symptoms, and HIV-related stigma. Among NCDs, frailty was most strongly linked to endocrine and metabolic disorders, renal and hepatic conditions, and respiratory conditions. One unexpected finding was the associations between prefrailty/frailty (FFP = 2–5) and internalized and clinic-based HIV stigma.

There is a lack of published studies from Central Asia on the prevalence of frailty among PLHIV, limiting regional comparisons. Nevertheless, the prevalence of frailty reported in this study is similar to that reported globally [12–16]. For example, the prevalence of prefrailty among PLHIV appears to be substantially high, with reported estimates of 38% in the United States, 39.1% in Spain, and 51.2% in Indonesia [27–30]. This may reflect the ongoing debate on accelerated versus accentuated aging in PLHIV, where HIV may either accelerate biological aging or increase the prevalence of age-related conditions without altering the rate of aging [29]. Furthermore, the scientific literature over the last 10–15 years suggests that the predictors of frailty and prefrailty are largely similar among the general population and PLHIV. These include older age, female sex, obesity, multimorbidity, and specific conditions such as chronic obstructive pulmonary disease (COPD), diabetes, stroke, coronary heart disease, and depression [31, 32].

Biologically, frailty is characterized by age-related dysregulation across multiple physiological systems, leading to a progressive decline in the body’s ability to maintain homeostasis in response to stressors. The underlying biological drivers include cellular and molecular hallmarks of aging, such as genomic instability, telomere attrition, epigenetic alterations, mitochondrial dysfunction, and altered intercellular communication [32]. While we did not specifically measure underlying biological mechanisms, they may contribute to frailty as observed in our sample. Notably, endocrine and metabolic disorders were among the most common conditions associated with prefrailty/frailty (FFP = 2–5) in our study. As observed in previous studies, these conditions, for example, diabetes, can lead to reduced insulin sensitivity, muscle catabolism, and sarcopenia, thereby contributing to frailty [33, 34].

Similarly, noninfectious lung diseases were associated with prefrailty/frailty (FFP = 2–5) in our sample. This aligns with previous studies demonstrating that conditions such as COPD are associated with frailty in both the general population and among PLHIV [35, 36]. PLHIV tend to have higher rates of COPD, likely due to a combination of persistent tobacco use and HIV-related factors such as chronic inflammation and immune activation [36]. In our study, 65% of the participants reported smoking daily, which is high. Although smoking could not be included in our regression models owing to the limited sample size, it is likely to have contributed to the observed frailty outcomes. Symptoms frequently documented in individuals with COPD, such as general exhaustion, muscle weakness, and shortness of breath, are consistent with the elevated prevalence of frailty reported in other studies [35].

An interesting finding is the association between HIV-related stigma and prefrailty/frailty (FFP = 2–5). HIV-related internalized stigma has been described as a chronic psychological stressor and has been associated with depressive symptoms, avoidance coping, and reduced social support among PLHIV, as demonstrated in prior studies, including Turan et al. [36]. These psychosocial consequences may contribute to frailty through behavioral and social pathways, such as decreased physical activity, poor nutrition, and reduced engagement in health maintenance behaviors [37]. Furthermore, PLHIV are often exposed to intersecting stigmas, including HIV-related stigma and ageism, which may exacerbate social withdrawal and physical and psychological isolation, thereby increasing vulnerability to frailty [37, 38]. A cross-sectional study in the U.S. involving 120 Latino PLHIV aged 50 years and older revealed that nearly half were frail [39]. Increased HIV-related stigma was significantly associated with greater odds of frailty, even after adjusting for income and comorbidities [39]. In contrast, we did not find associations between negative aging expectations and prefrailty/frailty, potentially because our study participants were relatively young.

Another noteworthy and concerning finding in our study was the association between prefrailty/frailty (FFP = 2–5) and HIV-related stigma within HIV care clinics. While our previous research revealed high levels of HIV-related stigma in Almaty’s primary healthcare settings, the expectation was that specialized HIV clinics would demonstrate greater sensitivity and competence in treating PLHIV [40]. However, findings from the Kazakhstan Stigma Index 2.0 indicate that stigma and discrimination persist even in HIV-specific healthcare settings [23]. This has serious implications, as stigmatizing experiences in care environments may discourage older PLHIV from engaging with healthcare services, potentially leading to poor treatment adherence and inadequate monitoring, both of which can accelerate frailty [41]. When trust and communication between patients and providers are compromised due to stigma, the result may be suboptimal care and a failure to address any type of health concern effectively. Furthermore, stigma-induced psychological distress, including depression and anxiety, may reduce physical activity and negatively affect overall health factors closely linked to frailty [41, 42].

Our pilot study is among the first to examine frailty among PLHIV in Kazakhstan and, to the best of our knowledge, in the broader Central Asian region. The use of both subjective and objective measures of frailty enhances the validity of our findings and enables a more comprehensive assessment of this condition in the study population. Nevertheless, several limitations should be acknowledged. First, this was a pilot follow-up study with a small sample size, which constrained our ability to perform more robust statistical analyses. The findings should therefore be interpreted with caution because of their statistical power and generalizability. The small sample size also limited the inclusion of key covariates, such as polypharmacy, as only a few respondents reported multiple medication uses. Furthermore, given its cross-sectional design, the study cannot establish the directionality of the observed associations. For example, psychosocial factors such as stigma and depressive symptoms, as well as multimorbidity, may act as both predictors and outcomes of frailty. Despite these limitations, the incorporation of clinical, behavioral, and objective frailty assessments provides a valuable foundation for future investigations on this important topic.

In conclusion, this study provides the first evidence on the prevalence and correlates of frailty among PLHIV in Kazakhstan and Central Asia, highlighting associations with multimorbidity, depressive symptoms, and HIV-related stigma. Although no statistically significant associations were observed between clinical HIV variables and frailty or prefrailty status in this study, future studies with larger sample sizes are needed to monitor and clarify these relationships among PLHIV. Importantly, our findings underscore the need to more comprehensively address frailty and aging in PLHIV to inform interventions that promote healthy aging. As PLHIV populations continue to age globally, this study supports a call to action for HIV and geriatric medicine to incorporate routine frailty screening and management into HIV care in order to better identify vulnerability and guide targeted interventions.

## Data Availability

The datasets used and/or analyzed during the current study are available from the corresponding author upon reasonable request.

## Abbreviations

PLHIV: People living with HIV
EECA: Eastern Europe and Central Asia
NCD: Noncommunicable disease
ART: Antiretroviral therapy
ACAPC: Almaty Centre for AIDS Prevention and Control
FFP: Fried frailty phenotype
CHS: Cardiovascular Health Study
PHQ-9: Patient Health Questionnaire-9
ERA-12: Expectations Regarding Aging, 12-item scale
AOR: Adjusted odds ratio
CI: Confidence interval
VIF: Variance inflation factor
COPD: Chronic obstructive pulmonary disease
